# An antibiotic agent pyrrolo[1,2-*a*]pyrazine-1,4-dione,hexahydro isolated from a marine bacteria *Bacillus tequilensis* MSI45 effectively controls multi-drug resistant *Staphylococcus aureus*†

**DOI:** 10.1039/c8ra00820e

**Published:** 2018-05-16

**Authors:** George Seghal Kiran, Sethu Priyadharsini, Arya Sajayan, Amrudha Ravindran, Joseph Selvin

**Affiliations:** Department of Food Science and Technology, Pondicherry University India; Department of Microbiology, Pondicherry University India josephselvinss@gmail.com

## Abstract

Sponge associated bacteria are a rich source of bioactive secondary metabolites. This study aims to isolate bacteria producing antimicrobial agents from a marine sponge, *Callyspongia diffusa*. A total of fifty-six bacteria were isolated and screened for antibacterial activity against multidrug resistant *S. aureus*. Based on the 16S rRNA sequence and phylogenetic analysis the antimicrobial producer strain MSI45 was identified as a novel *Bacillus tequilensis*. The culture conditions of strain MSI45 were optimized to enhance the yield of the antimicrobial compound. The antimicrobial compound was purified using a silica gel column chromatography and high performance liquid chromatography. On the basis of spectroscopic analysis such as FT-IR, NMR and GC-MS, the bioactive metabolite was identified as pyrrolo[1,2-*a*]pyrazine-1,4-dione,hexahydro. The extracted compound MSI45 showed a potent inhibitory effect on multidrug resistant *S. aureus* with an MIC of 15 ± 0.172 mg L^−1^ and MBC of 20 ± 0.072 mg L^−1^. The compound was non-hemolytic and showed high antioxidant activity. The antioxidant activity may increase the efficacy and safety of the molecule in drug development. Hence, this compound produced by *Bacillus tequilensis* MSI45 could have potent antimicrobial and antioxidant activity against *S. aureus* infection.

## Abstract 2

A new antibiotic agent from sponge associated marine bacteria.

## Graphical abstract

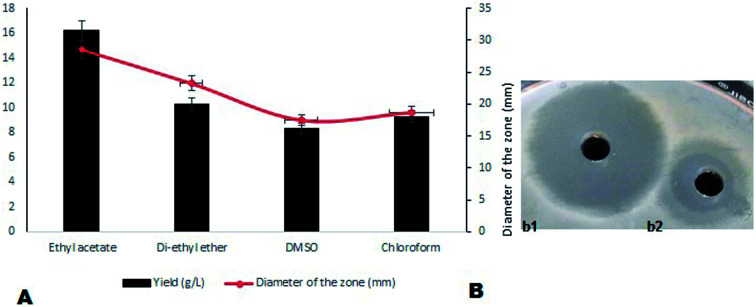

## 1. Introduction

The emergence of multidrug resistant (MDR) bacterial pathogens has posed a major threat to the public as the bacteria develop resistance against existing antimicrobial agents.^1^ Hence, the screening of a new class of anti-infective compounds is essential to treat major infectious diseases. Microbes surviving in profoundly hostile conditions usually produce secondary metabolites to repress the settlement of ecological competitors.^2^ Marine sponges and their microbial symbiosis is a unique ecological phenomenon which serves as the reservoir of novel bioactive molecules.^3^ More than 250 novel compounds are reported every year from marine sponges.^4^ Moreover, sponge derived metabolites are important substances which act as anti-inflammatory and anticancer agents.^5^ Marine sponge associated *Bacillus* can produce anticholinesterase enzyme which can degrade acetylcholine in the brain.^6^*Streptomyces* spp. is the most widely explored bacterial species from Indian coast for the production of antimicrobial compounds.^3^ Progress in genome mining and metagenomic approaches has led to the manipulation of sponge associated microbial diversity and exploration of complex biosynthetic pathways of marine natural products.^7^ Different classes of compounds such as bioactive fatty acids, alkaloids, terpenes, sterols, cyclic peptides, peroxides, and amino acid derivatives are reported to be produced by sponges and their associated microbes.^8–16^ Bioactive peptides derived from the sponges are of non-ribosomal origin and contain unusual amino acids.^3,17^*Bacillus* sp. are known for their high probiotic potential and more than 200 antibiotics have been produced by *Bacillus* and these antibiotics differ both in activity and structure.^18^ They are well known producers of antimicrobial compounds such as bacteriocin like inhibitory substances (BLIS) iturin, surfactin, fengycins and bacteriocins.^19^ Ribosomally active antibiotics showed decreased virulence secretion in *S. aureus* when compared to cell wall active antibiotics such as beta lactams which increases the exotoxin production.^20^ The greatest issue of multidrug resistance is associated with the Gram-positive bacteria such as *Pneumococci*, *Enterococci* and *Staphylococci* among which the *S. aureus* is of much concern because of its virulence, pathogenicity, adaptability in diverse habitats, ability to cause severe infections and mortality. Even when new antibiotics are used against *S. aureus* it develops effective mechanism to neutralise them. Hence, this study was focused to develop antimicrobial agent from marine bacteria against *S. aureus*. The *S. aureus* used in this study was resistant to all antibiotics including vancomycin and hence, we aimed to screen the antibacterial compound from a marine sponge associated bacteria. In this study, we report a novel *Bacillus tequilensis* MSI45 isolated from a marine sponge which exhibited potent antimicrobial and antioxidant activities against MDR *S. aureus*. The compound was structurally characterized by FT-IR, GC-MS and NMR. The active compound was identified as pyrrolo[1,2-*a*]pyrazine-1,4-dione,hexahydro.

## 2. Materials and methods

### 2.1 Sponge collection and isolation of bacteria

The marine sponge *Callyspongia diffusa* was collected by scuba diving at Vizhinjam (Kerala) coast located in southwest coast of India at a depth of 1 meter and 1500 meters away from the sea shore. The collected sponge was rinsed with sterile seawater to remove any adhered exogenous material and processed for the isolation of microorganisms. The rinsed sponge was then cut into small pieces using a sterile blade. A few drops of sterile distilled water were added, and the pieces were crushed using a sterile mortar and pestle. Then, it was serially diluted and plated on to Zobell Marine Agar (these ZMA, Himedia plates streaked with samples were incubated at 30 °C for 48 h). Morphologically distinct colonies were selected and then re-streaked till the pure cultures were obtained.^21^

### 2.2 Screening for antimicrobial activity

The antibiotic resistance pattern of clinical isolates (13 *S. aureus* phenotypes) were collected from the clinical laboratories (Puducherry) and were screened against antibiotics (Himedia) such as ampicillin 10 mcg per disc, azithromycin 15 mcg per disc, chloramphenicol 30 mcg per disc, ciprofloxacin 5 mcg per disc, erythromycin 15 mcg per disc, gentamicin 10 mcg per disc, kanamycin 30 mcg per disc, mecillinam 10 mcg per disc, penicillin-G 10 units per disc and vancomycin 30 mcg per disc as per the Clinical & Laboratory Standards Institute: CLSI Guidelines (2013). The phenotypes (6) showed complete antibiotic resistance was selected for the activity screening.

The isolated colonies were cultivated on Zobell marine broth (Himedia) at 28 °C for 96 h at 200 rpm. The cell free supernatant (CFS) was obtained by centrifugation (Eppendorff) at 10 621 × *g* for 15 min and was used for the screening of antimicrobial activity by agar well diffusion assay.^22^ Muller Hinton agar (MHA) plates were prepared and overnight grown culture of *S. aureus* was swabbed on the surface of MHA plates. Then wells were made using a sterile steel cork borer and the wells were filled with 50 μl of cell free supernatant (CFS) and incubated at 37 °C for 24 h. Later the plates were inspected for zone formation.

### 2.3 Identification of antimicrobial producer

The potent antimicrobial producer MSI45 was morphologically and physiologically characterised by the Logan and Berkley's method.^23^ Genomic DNA of the isolate MSI45 was extracted using a genomic DNA extraction kit (sigma). Universal 16S rRNA eubacterial primer (5′-GAGTTTGATCCTGGCTCAG-3′; 5′-AGAAAGGAGGTGATCCAGCC-3′) was used for the amplification of DNA. The 16S rRNA gene sequence obtained from the isolate MSI45 was compared with other bacterial sequences using NCBI mega BLASTn (http://blast.ncbi.nlm.nih.gov/Blast.cgi) for their pair wise identities. Multiple alignments of these sequences were carried out by Clustal W 1.83 version of EBI (www.ebi.ac.uk/cgi-bin/clustalw/) with 0.5 transition weight. Phylogenetic trees were constructed in MEGA 7.0 version (www.megasoftware.net) using a maximum parsimony algorithm.

### 2.4 Screening of culture medium for the production of antimicrobial compound

To study the effect of culture media on the production of antibacterial compounds 50 μl from overnight grown culture of MSI45 was inoculated into 100 ml of different media such as nutrient broth supplemented with 2% NaCl, Luria Bertani broth, modified marine broth, starch casein broth, and Zobell marine broth (Himedia). Each culture medium was then incubated at 28 °C for 72 h. After incubation cell free supernatant was obtained by centrifugation at 10 621 × *g* for 15 min. Then antibacterial activity of the CFS separated from different media was determined using a agar well diffusion assay as mentioned above.

### 2.5 Optimization of process parameters

In addition to the production media, various carbon, nitrogen sources and salt concentrations were optimized. Carbon sources used in the optimization process include 1% each of starch, glucose, sucrose and lactose and nitrogen sources used were ammonium nitrate, beef extract peptone and yeast extract. The effective carbon and nitrogen sources were further optimized by varying the concentration of chosen carbon and nitrogen source in increasing increments from 1–5%. In addition, the effect of cultural conditions like varying salt concentration of 1–4% NaCl and different temperature ranging between 20 to 40 °C and pH 4.0–9.0 were optimized during the process development. The optimum cultural conditions and nutritional supplements required for optimum growth and enhance the yield of antimicrobial compound was determined by a well diffusion assay as described above.

### 2.6 Screening of solvent for the extraction of antimicrobial compound MSI45

The CFS of MSI45 was mixed with an equal volume of six different solvents such as chloroform, ethyl acetate, ethanol, dimethyl ether, dimethyl sulfoxide and methanol in separate extraction flasks and kept for overnight incubation at 4 °C. The solvents were then evaporated in a rotary evaporator (Yamato) and the remaining residues were concentrated in vacuum concentrator (Labconco) and lyophilised in a lyophilizer (Yamato). The lyophilised compounds were dissolved at a concentration of 50 mg L^−1^ and from this 100 μl of the compound MSI45 was tested for antimicrobial activity using a well diffusion assay.

### 2.7 Purification of antimicrobial compound

The lyophilized compound from MSI45 was dissolved in DMSO at a concentration of 100 μg ml^−1^ and subjected to silica gel thin layer chromatography (TLC) using chloroform methanol and water (60 : 30 : 10) as the solvent system. The lyophilized compound was purified by using a silica gel column chromatography with chloroform, and methanol (6 : 4 v/v) was used as the eluting solvent. The purified fractions were checked for bioactivity using a well diffusion assay against *S. aureus*. Further, the active fractions were purified by a HPLC using the same solvent system used for column chromatography with a flow rate of 0.5 ml min^−1^. The purified active fraction of MSI45 was checked for hemolysis using 5% of sheep blood agar plate.^24^ The purified active compound was subjected to a Fourier transform infrared spectrophotometer (Perkin Elmer, USA). A constituent of the metabolite was investigated using a gas chromatography (GC) (Perkin Elmer Autosystem XL GC-TurboMass, USA). The data was processed by a GC-MSD, Chemstation column conditions were programmed as column oven temperature 150 °C (4 min) 4 °C min^−1^, temperature of inject port 250 °C and detector port 280 °C. The peaks of the gas chromatography were subjected to mass spectral analysis and the spectra were analyzed by NIST MS search (version 2.0). ^1^H and ^13^C NMR spectra was acquired by dissolving the purified compound in deuterated DMSO at a concentration of 10 mg ml^−1^ and analyzed on a Bruker Avance II 500 Spectrophotometer (Bruker BioSpin AG, Switzerland) at 22 °C.

### 2.8 Determination of minimum inhibitory concentration (MIC)

The MIC of MSI45 was determined by a broth microdilution method in 96-well microtiter plates according to the EUCAST standards for testing antimicrobial susceptibility^25^ with suitable modifications. Briefly, about 10^8^ cells per ml of bacterial suspension was subsequently diluted to 1 : 300 in tryptic soy broth. Different concentrations (10, 20, 30, 40, 50, 100, 150, 200 mg L^−1^) of MSI45 extract were dissolved in water and were used to determine MIC. To the sterile flat bottom 96-well plate 200 μl of the diluted bacterial suspension and varying concentration of MSI45 was added and the cultures were incubated at 37 °C for 24 h. Wells with *S. aureus* alone served as positive control and wells with water served as negative control. The MIC was determined as the lowest concentration of MSI45 for which no visible bacterial growth was observed after 24 h of incubation. To determine the minimum bactericidal concentration (MBC), the CFU ml^−1^ was further evaluated in broth culture from wells without visible growth. All the experiments were performed in triplicates and repeated several times. The lowest concentration of the extract that showed reducing number of viable cells by at least three orders of magnitude was considered as MBC according to the recommendation of the European Committee for Antimicrobial Susceptibility Testing (EUCAST) of the European Society of Clinical Microbiology and Infectious Diseases (ESCMID).^26^

### 2.9 Time-kill assay

Time-kill studies were performed according to the method described.^27^ Exponential phase colonies of *S. aureus* cells (10^6^ CFU ml^−1^) were allowed to grow in a 6 deep well plates in triplicates and treated with MSI45 of different concentration of 1× MIC, 2× MIC, 4× MIC, 8× MIC to a final concentration of 80 mg L^−1^ and incubated at 37 °C, for 12 h and then 200 μl of respective samples were serially diluted in 10 mM MgSO_4_ and CFU ml^−1^ of *S. aureus* were checked in varying time intervals by plating on TSA plates (tryptic soya agar) for enumeration of colony forming units (CFU).

## 3. Antioxidant activity

Antioxidant activity of the compound from MSI45 was determined using a DPPH radical scavenging assay,^28^ with necessary modifications. 0.2 ml of different concentrations of the compound MSI45 (25, 50, 75, 100, 125 and 150 μg ml^−1^) were added to 2.4 ml of 0.1 mM DPPH methanol solution. The assays were performed in triplicates, ascorbic acid served as the positive control and methanol was used as a blank. The assay mixture was vortexed and incubated in dark at 25 °C for 60 min. The process of decolourization was recorded at 520 nm using a Shimadzu UV-VIS spectrophotometer. The percentage of radical scavenging was calculated using the below mentioned formula.AA (%) = [(Abs._control_ − Abs._sample_)/Abs._control_] × 100

### 3.1 MTT assay

Mouse embryo fibroblast cell line was purchased from NCCS, Pune and the cells were maintained in DMEM – high glucose medium. To the 96-well plate 200 μl of cell suspension was seeded at a required cell density (20 000 cells per well) and the cells were allowed to grow for about 12 h. To these cells MSI45 of different concentrations 10, 25, 50, 100, 200 mg L^−1^ was added and the plates were incubated at 37 °C in a humidified atmosphere (5% CO_2_ and 95% air). After incubation, MTT reagent was added to the plates to make a final concentration of 0.5 mg ml^−1^ and the plates were again incubated for 3 h. The MTT reagent was removed and then 100 μl of solvent (DMSO) was added. The absorbance was read on a microtitre well plate spectrophotometer at 570 nm and 630 nm (Biotek Instruments, USA). The values of the percentages of cell viability were plotted against MSI45 concentrations, and CC_50_ was determined.

## 4. Results

### 4.1 Isolation and characterisation of sponge associated bacteria

**Fig. 1:**
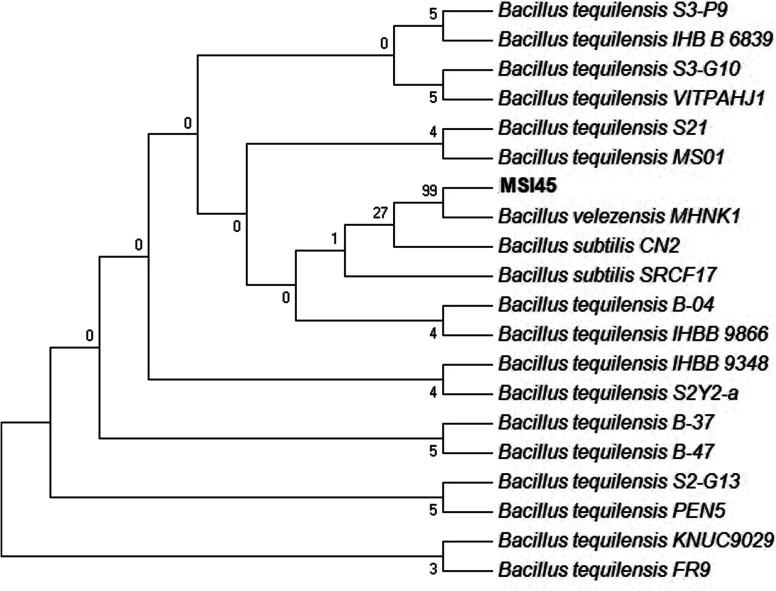
Phylogenetic tree of MSI45 shows representatives of *Bacillus tequilensis*. The tree was inferred using maximum parsimony method.

A total of 56 marine bacteria were isolated from the marine sponge *Callyspongia diffusa* and were screened for antimicrobial activity against *S. aureus*. The pathogen *S. aureus* used in this study was resistant to all antibiotics tested which include vancomycin, penicillin, gentamycin, kanamycin, oxacillin, streptomycin and chloramphenicol. Among the isolates screened MSI45 showed antibacterial effect with a clear zone of inhibition (22 mm). The selected isolate MSI45 was characterized as Gram positive, non-motile, non-haemolytic, and catalase, protease and phospholipase negative. The isolate MSI45 was found to be sensitive to all the antibiotics tested such as ampicillin, chloramphenicol, tetracycline, and oxacillin. Taxonomic affiliation of the isolate MSI45 based on 16S rRNA sequencing revealed the isolate has closest association with *Bacillus tequilensis* (Fig. 1). The BLASTn analysis showed 99% identity with *B. tequilensis*, *B. velezensis* and *B. subtilis*. Based on the biochemical characteristics and halophilic nature of MSI45, the strain was tentatively identified as *B. tequilensis.* This work is the first report to identify *B. tequilensis* from a marine sponge.

### 4.2 Optimization and extraction of the antimicrobial compound MSI45

**Fig. 2:**
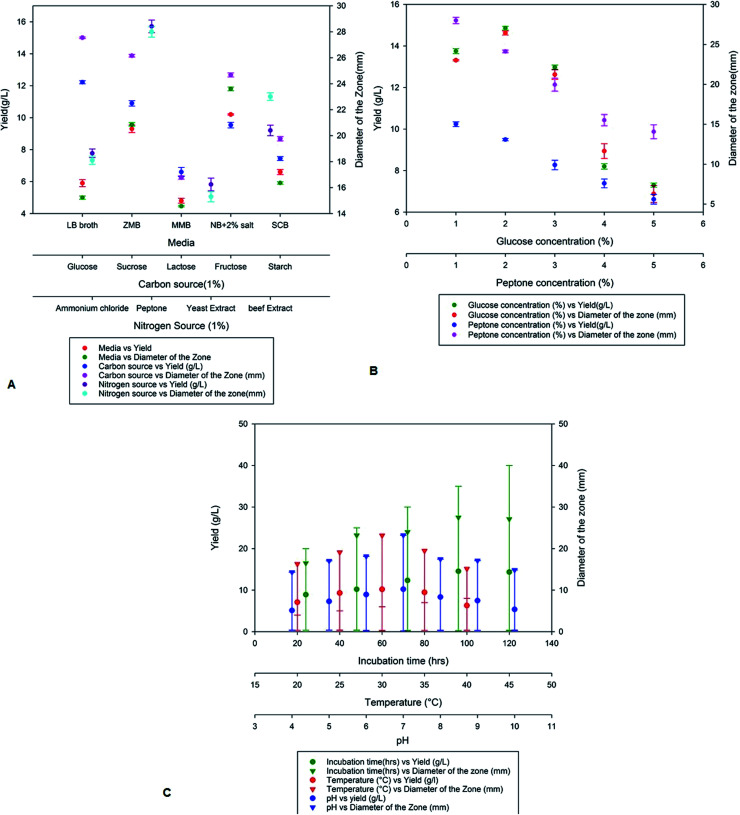
Optimization of nutritional and physical parameters for the growth of MSI45 and production of antimicrobial compound.

On optimization of various production media nutrient agar supplemented with 2% NaCl showed high production of antimicrobial compounds when compared to Zobell marine broth, marine modified broth, LB broth and starch casein broth. A maximum yield of 10.2 g L^−1^ was obtained on the nutrient broth media supplemented with 2% NaCl with a zone of inhibition of 23.2 mm (Fig. 2a). The yield of antimicrobial compound MSI45 was further improved on addition of various carbon and nitrogen sources. We observed that the media supplemented with 1% each of glucose and peptone increases the yield of the compound MSI45. The effect of different concentration of carbon and nitrogen sources on antimicrobial activity showed 2% glucose and 1% peptone was optimum for the growth and for the yield of 15 g L^−1^ with a diameter of zone of inhibition of 28 mm (Fig. 2b). Physical parameters such as temperature, incubation time and pH were also optimized. We observed a temperature of 30 °C with an incubation time of 96 h and a pH of 7.0 to be suitable for the yield of antimicrobial compound (Fig. 2c).

### 4.3 Solvent optimization

**Fig. 3:**
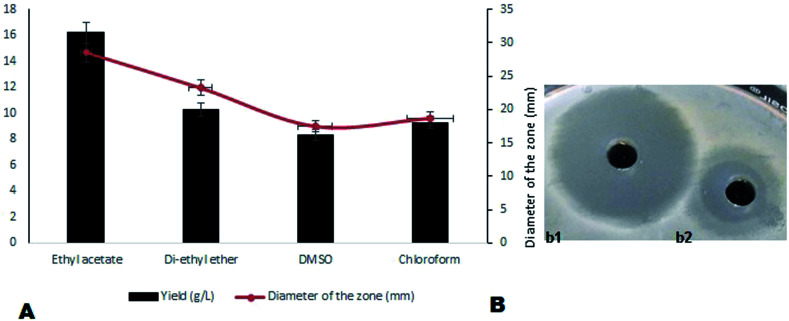
(A) The solvents used for extraction and it was observed that ethyl acetate enhances the yield of antimicrobial compound and (B) (b1) shows the zone of inhibition of optimized MSI45 against MDRSA and (b2) shows the zone of inhibition before optimization of the compound MSI45.

On optimization of solvents for the extraction of compound MSI45, ethyl acetate was found to be the most suitable solvent followed by diethyl ether and DMSO. A maximum yield of 16.2 g L^−1^ of antimicrobial compound was recovered from the media using ethyl acetate. The activity of the ethyl acetate extracted compound was tested by a well diffusion assay and the results obtained are shown in Fig. 3. The extracted compound was found to be non-haemolytic and therefore it can be used for clinical applications.

### 4.4 Determination of MIC and MBC

**Fig. 4:**
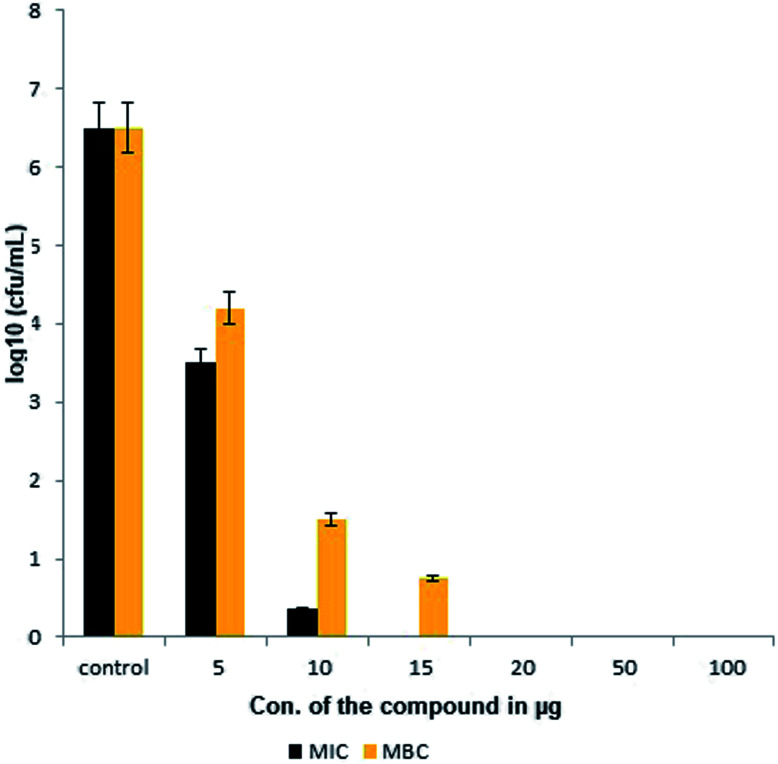
Minimal inhibitory and minimal bactericidal concentration of the compound MSI45.

The extracted compound MSI45 showed potent inhibitory effect on MDRSA strain with an MIC of 15 ± 0.172 mg L^−1^ and MBC of 20 ± 0.072 mg L^−1^ (Fig. 4). The MIC and MBC ratio was found to be 0.75 and hence the compound was identified as bactericidal.

### 4.5 Time kill assay

**Fig. 5:**
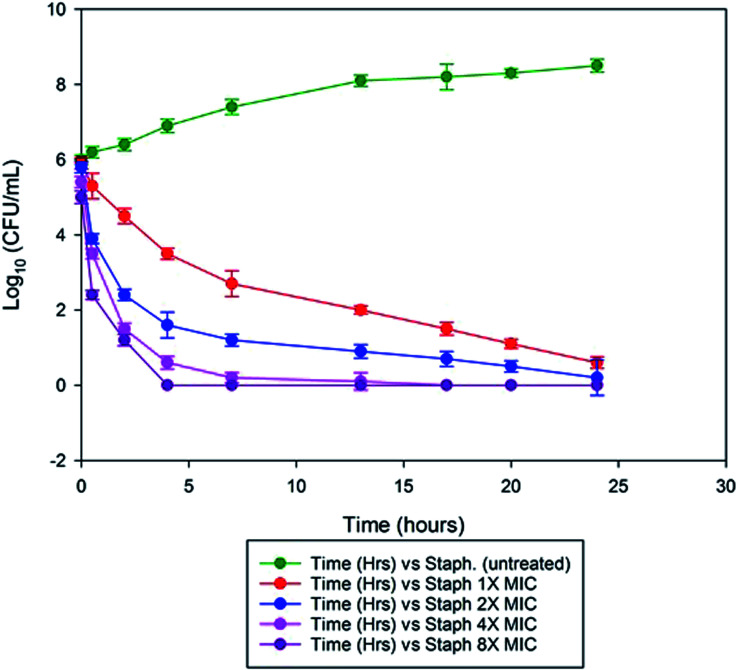
Time kill assay showed the inhibition of the *S. aureus* by the extracted compound at 1× MIC, 2× MIC, 4× MIC and 8× MIC. At 1× MIC the complete inhibition was achieved within 24 h of incubation and when MIC was increased to 4× and 8× the bactericidal effect was noticed within 2 h of incubation.

We observed the minimal inhibitory effect of MSI45 against *S. aureus* was 15 ± 0.172 mg L^−1^. In the time kill assay, the killing rate of MSI45 increased with increase in the concentration of MIC. At 1× MIC of MSI45 the complete killing of *S. aureus* cells was achieved at 24 h, at 4 and 8× MIC the bactericidal effect was well noticed within 2 h of incubation (Fig. 5). The CFU was reduced by 7 log units indicating the fast killing effect of MSI45. The time kill kinetics showed a dose dependent bactericidal effect on MDRSA, when the dose of the compound was increased the death of the *S. aureus* cells was achieved indicating the potent antimicrobial effect of the extract MSI45.

### 4.6 Characterization of antimicrobial compound MSI45

**Fig. 6:**
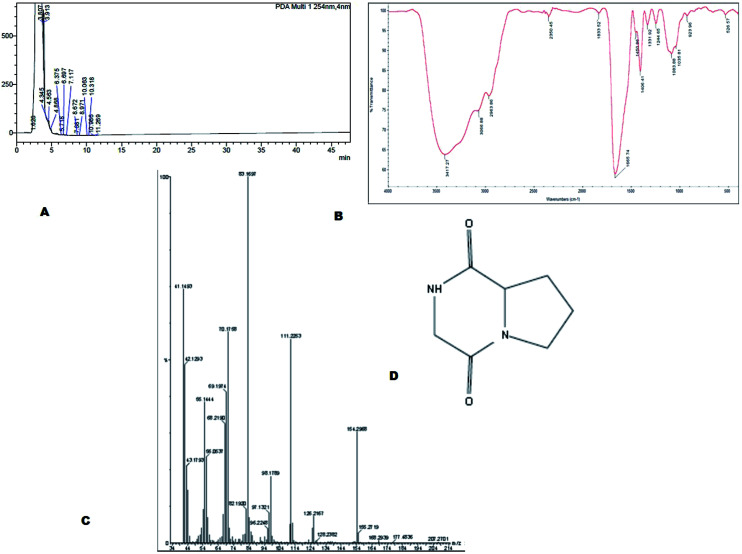
(A) Purification of the compound using HPLC and (B) FTIR analysis shows the functional groups of the compound MSI45 (C) mass spectral analysis of the compound using GCMS and (D) predicated structure of the compound MSI45 as pyrrolo[1,2-*a*]pyrazine-1,4-dione,hexahydro.

The bioactive compound MSI45 was purified by a HPLC which showed an elution peak at the retention time of 3.11 min (Fig. 6A). The eluted active compound from MSI45 was found to be non-hemolytic on blood agar plates (ESI Fig. 1†). The FTIR spectrum of the compound MSI45 showed characteristic functional groups at 3417–3060 cm^−1^ corresponding to amine N–H stretch, peak at 2963 cm^−1^ corresponding to alkyl C–H stretch, carbonyl group at 1665 cm^−1^ and C–O bonds at 1450–1244 cm^−1^ respectively (Fig. 6B). The GC-MS analysis was performed to determine the active molecule of MSI45. The analysis showed presence of four compounds in the sample on comparison of the mass spectra, molecular weight, retention time and molecular formula with the NIST library. The spectra showed a major peak at 16.9 min the mass spectrum analysis showed it has a molecular weight of 154 Da with a molecular formula of C_7_H_10_O_2_N_2_ (pyrrolo[1,2-*a*]pyrazine-1,4-dione,hexahydro) (Fig. 6C). The peak area corresponds to the quantity of the compound present in the sample. The ^1^H-NMR spectrum (400 MHz, DMSO) showed signals at *δ*_H_ 1.8–2.3 corresponding to 4H, and also a (2H) peak at 3.5–3.6 and signals at 3.8 (d, 1H), 3.9 (d, 1H), 4.1 (dd, 1H) and 7.1 (1H, S) The ^13^C NMR spectrum showed signals at *δ* 22.23, 28.93, 45.78, 46.01, 58.04, 164.63, 169.51 (ESI Fig. S2†). The NMR, FT-IR and GC-MS spectral data helped in identification of the compound MSI45 as pyrrolo[1,2-*a*]pyrazine-1,4-dione,hexahydro.

### 4.7 DPPH scavenging assay

**Fig. 7:**
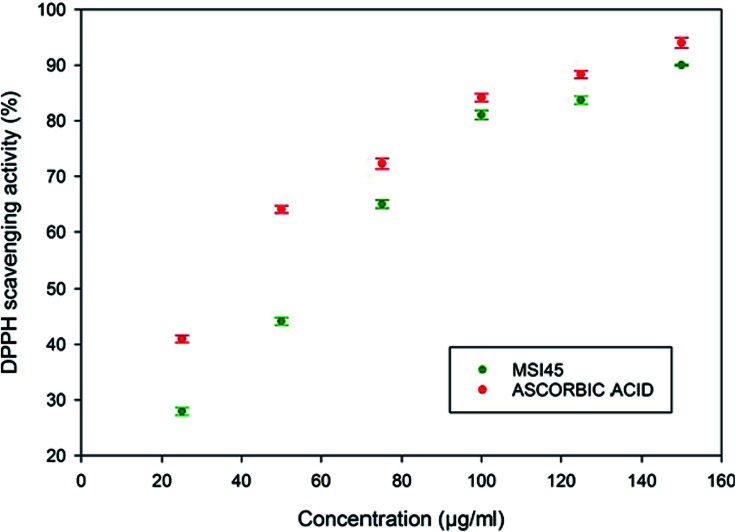
Antioxidant activity of the compound MSI45 using DPPH assay. The assay shows the antioxidant activity increases with increase in the concentration of the compound.

The antioxidant activity of the compound MSI45 was tested using a DPPH scavenging assay. The free radical scavenging activity of the compound increased in a dose dependent manner. The activity increased from low concentration of 25–125 μg ml^−1^. The scavenging activity of the compound was found to be 27.95 ± 0.68 for 25 μg and 90 ± 0.12 for 125 μg ml^−1^ (Fig. 7). The antioxidant activity of the compound MSI45 was much similar when compared to the standard ascorbic acid at higher concentration of 75–100 μg ml^−1^. This trend shows an increase in the concentration of the compound MSI45 has a significant higher antioxidant activity.

### 4.8 Cell line studies

**Fig. 8:**
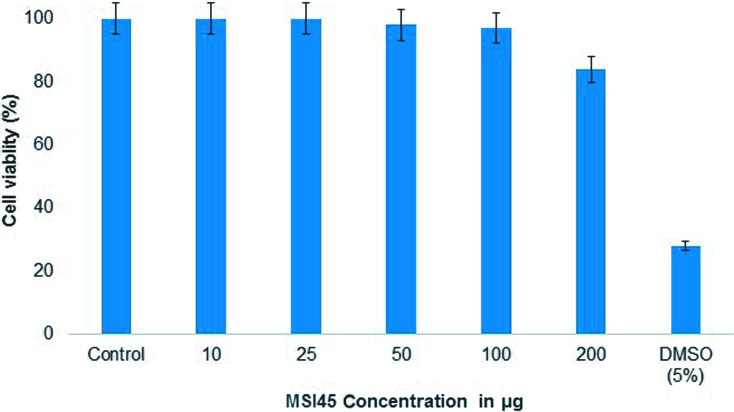
The MTT assay showed nontoxic nature of the compound MSI45.

MTT assay was performed to determine the toxicity of the compound MSI45 to the cell line. The mouse embryo fibroblast cell line was treated with varying concentration of the compound MSI45 and the results obtained showed high viability rates as compared to the control. On an average of the triplicate experiment, 97% viability was achieved up to 100 mg L^−1^ and 84% viability in 200 mg L^−1^ (Fig. 8). Thus, the obtained CC_50_ values showed that the antimicrobial compound MSI45 can be suitable for systemic application.

## 5. Discussion

Marine sponges are the richest source of antimicrobial compounds such as terpenoids, peptides, polysaccharides, polyketides, fatty acids, steroids, alkaloids and phenolic compounds. The alkaloids produced by the sponges have nitrogen containing heterocyclic compounds which include bromopyrrole, pyrroloiminoquinone and alkylpiperidine alkaloids.^29^ The sponge *Callyspongia diffusa* used in this study for bacterial isolation was known for the production of antimicrobial compounds^30^ and the bacteria isolated from the sponges were reported for the production of antifouling, biosurfactant and antibacterial compounds.^31^ Most of the bacteria associated with sponges were endosymbiotic and provide a major source of bioactive molecules. Marine *Bacillus* species were well known for the production of bioactive compounds especially macrolactin^32^ and biosurfactants. In this study a total of 56 bacteria were isolated from the marine sponge and they were screened for the antibacterial activity against multidrug resistant *S. aureus*. We identified and isolated a novel *B. tequilensis* MSI45 which showed inhibitory effect against *S. aureus*. A probiotic strain of *B. tequilensis* FR9 has been isolated from the gastrointestinal tract of chicken without any haemolytic activity and was found to be tolerant in acid and bile test.^33^ A sponge isolate *Bacillus* SEB32 produced antimicrobial agent in the Zobell marine broth supplemented with 35% NaCl on 9^th^ day of incubation.^34^ The isolate used in this study *B. tequilensis* MSI45 produced a antimicrobial compound on 4^th^ day of incubation with an inhibition zone of 28 mm. On optimization of solvents for the extraction of bioactive molecule ethyl acetate was found to be most suitable and recovered the maximum yield of the compound 16.2 g L^−1^. Based on FT-IR, GC-MS and NMR characterization of the bioactive molecule from *B. tequilensis* it was observed that the active molecule was pyrrole with its substitute as pyrrolo[1,2-*a*]pyrazine-1,4-dione,hexahydro. The peaks and the functional groups in FT-IR were similar to the study reported.^35^ Pyrrole was known for various therapeutic applications and has been used as antibiotics, antitumour, antifungal, anti-inflammatory and cholesterol reducing drugs. Pyrrole has also been the ability to inhibit HIV-1 viruses and DNA polymerases and protein kinase activity.^36–38^ Pyrrole along with its substitute, pyrazine protects the seizures and is used as a potent anticonvulsant drug.^39^ A *Streptomyces* strain isolated from mangrove soil has been reported to produce pyrrolo[1,2-*a*] pyrazine-1,4-dione,hexahydro showed antioxidant property of 32% in 1 mg ml^−1^ of the compound^40^ whereas the antioxidant property of the compound produced by MSI45 was much higher of 81% in 0.1 mg ml^−1^. In the time kill assay the complete inhibition of *S. aureus* was achieved at 1×, 4× and 8× MIC within 24 and 2 h of incubation. A study by Li *et al.*^41^ reported hexahydropyrrolo[1,2-*a*]pyrazine-1,4-dione as an algicidal agent against *Microcystis aeruginosa*.

## 6. Conclusions

In this study a marine sponge *C. diffusa* associated bacteria *B. tequilensis* was isolated, identified and optimized to produce antibiotic compound that acted on MDRSA. The spectroscopic analysis revealed the bioactive metabolite as a pyrrolo[1,2-*a*]pyrazine-1,4-dione,hexahydro. This molecule was non-hemolytic and showed antioxidant activity. Therefore, it was concluded that the molecule produced by *B. tequilensis* MSI45 can be developed as an effective antibiotic against infections caused by MDRSA.

## Author contributions

JS designed, monitored and written the paper. GSK guided all experimental works. SP and AS performed lab experiments.

## Conflicts of interest

There are no conflicts of interest to declare.

## Supplementary Material

RA-008-C8RA00820E-s001
