# Supplementary material for: An antibiotic agent pyrrolo[1,2-a]pyrazine-1,4-dione,hexahydro isolated from a marine bacteria Bacillus tequilensis MSI45 effectively controls multi-drug resistant Staphylococcus aureus
Source: RSC Adv. 2018 May 16;8(32):17837–46. doi: 10.1039/c8ra00820e (PMC9080480; doi:10.1039/c8ra00820e)
Supplement: RA-008-C8RA00820E-s001 [file RA-008-C8RA00820E-s001.pdf]

Electronic Supplementary Material (ESI) for RSC Advances.
This journal is © The Royal Society of Chemistry 2018

Supplementary Figure legends

Supplementary Figure 1) Non- Hemolytic nature of the compound MSI45 on blood agar plates.

Supplementary Figure 2) ^{1}H and ^{13}C NMR spectrum of the compound MSI45 showing characteristic peaks at δH 1.8 -2.3 and at (2H) peak at 3.5 – 3.6 and signals at 3.8 (d, 1H), 3.9 (d, 1H), 4.1 (dd, 1H) and 7.1 (1H, S) The ^{13}C NMR spectrum showed signals at δ22.23, 28.93, 45.78, 46.01, 58.04, 164.63, 169.51 showing the compound as pyrrolo[1,2-A]pyrazine-1,4-dione,hexahydro

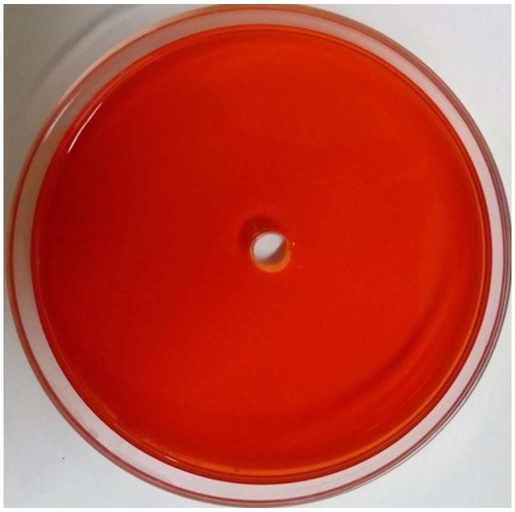

Figure S1.

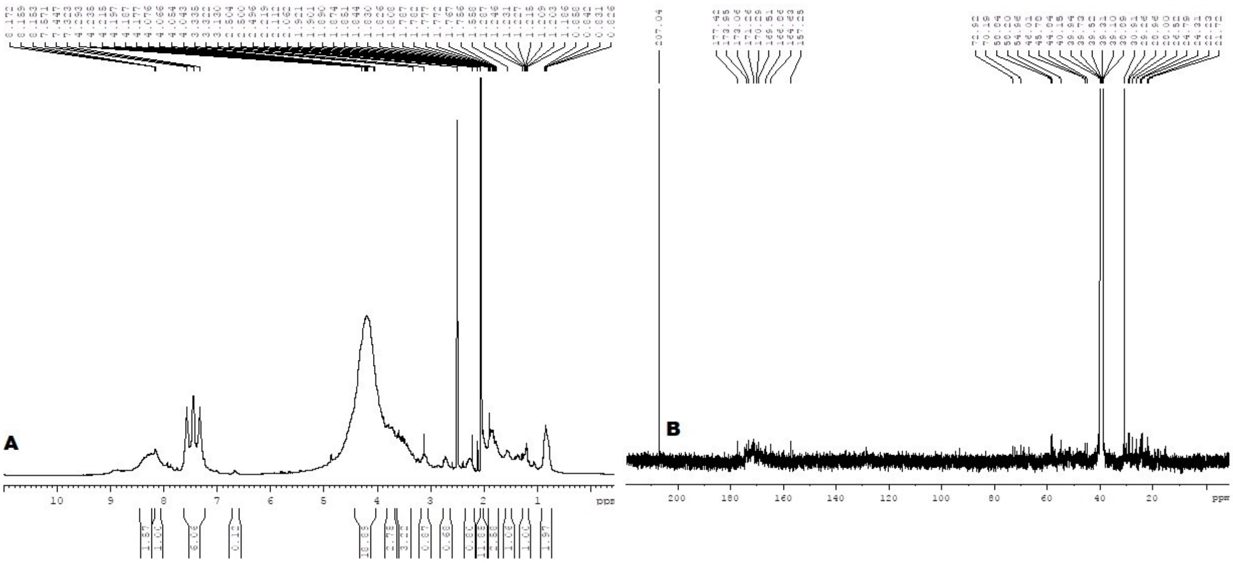

Figure S2.
